# Relativistic Prolapse-Free Gaussian Basis Sets of Double- and Triple-ζ Quality for *s*- and *p*-Block Elements: (aug-)RPF-2Z and (aug-)RPF-3Z

**DOI:** 10.1021/acs.jctc.4c01211

**Published:** 2024-11-14

**Authors:** Julielson dos Santos Sousa, Eriosvaldo Florentino Gusmão, Anne Kéllen de Nazaré dos Reis Dias, Roberto Luiz Andrade Haiduke

**Affiliations:** Department of Chemistry and Molecular Physics, São Carlos Institute of Chemistry, University of São Paulo, C.P. 780, São Carlos, São Paulo 13560-970, Brazil

## Abstract

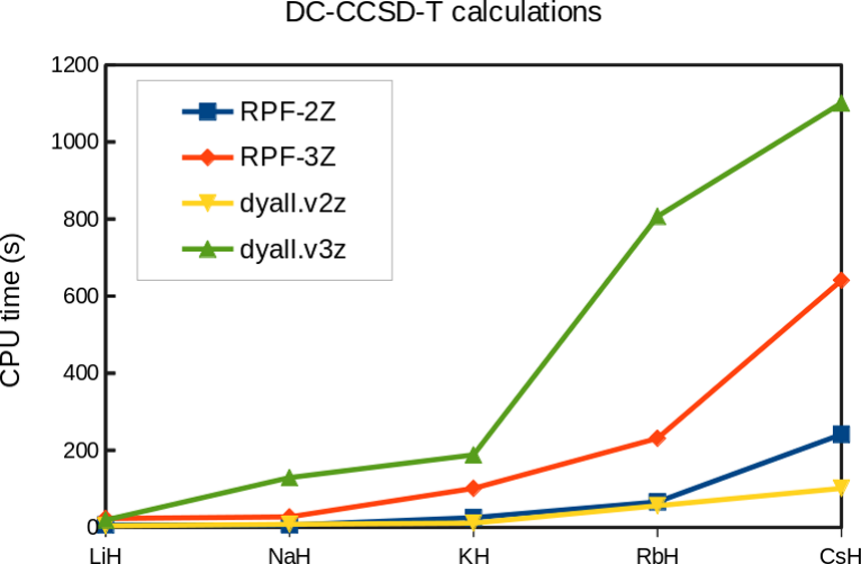

This study presents two new relativistic Gaussian basis sets without variational prolapse of double- and triple-ζ quality, RPF-2Z and RPF-3Z, along with augmented versions including additional diffuse functions, aug-RPF-2Z and aug-RPF-3Z, which are available for all *s* and *p* block elements from Hydrogen to Oganesson. The exponents of the Correlation/Polarization (C/P) functions are obtained from a polynomial version of the generator coordinate Dirac–Fock method (p-GCDF). The choice of C/P functions was guided by multireference configuration interaction calculations with single and double excitations (MR-CISD) based on a valence active space. Finally, calculations of fundamental properties done for atomic and molecular systems (bond lengths, vibrational frequencies, dipole moments, and electron affinities) ensure the expected quality of these new basis sets, which may also exhibit some computational efficiency advantages. Additionally, the prolapse-free feature of these sets must provide a reliable description of properties more dependent on core electron distributions, as well.

## 1 Introduction

The proposal of the Dirac equation in 1928,^1^ which unified the quantum mechanics with the special relativity theory, motivated the practical implementation of these ideas in electronic structure studies and gave origin to an important discussion regarding relativistic effects in chemistry.^2^ In this context, the four-component formalism provides one of the most reliable treatments of relativistic effects in quantum mechanics. Among other aspects, this remarkable advance evidenced the need for developing new sets of relativistic basis sets for four-component calculations,^3−40^ which are also available for the usage with systems containing heavy and superheavy elements.

Unfortunately, most of these sets may present a problem known as variational prolapse,^17−19^ which is often characterized by obtaining Dirac–Fock–Roothan (DFR) electronic energies lower than the corresponding reference values from numerical methods (NDF—Numerical Dirac–Fock). However, variational prolapse may also be observed in basis sets that provide energies larger than the exact values. This basis set issue occurs due to an improper representation of the spinors around the nuclear region.^17−19,41^ In other words, variational prolapse is considered a basis set incompleteness issue^19^ that may be detected in general situations by obtaining energy increments along the addition of tight functions.^41^ Hence, this basis set deficiency should be addressed, as one is interested in properties more dependent on inner electron distributions, for instance. Thus, aiming the generation of reliable and competitive basis set alternatives, Haiduke and da Silva provided large relativistic prolapse-free sets of Gaussian functions (LRPFs)^26,27^ by means of the polynomial version of the Generator Coordinate Dirac–Fock (p-GCDF) method,^42^ while Teodoro and collaborators extended this approach to treat superheavy atoms.^28^ Soon after, these sets were complemented with Correlation/Polarization (C/P) functions for s and p block elements,^43^ as well as for those atoms of d and f blocks.^44,45^ These sets were named quadruple-ζ quality relativistic prolapse-free sets (RPF-4Z).

Recently, small- and medium-size relativistic prolapse-free sets of primitive Gaussian functions (SRPFs and MRPFs, respectively) were also generated with the p-GCDF protocol for all elements from Hydrogen until Oganesson.^46−48^ Hence, the Dirac–Fock-Coulomb energies presented in these studies are never below the numerical reference values, and the addition of tight functions proves that there is not any evidence of variational prolapse. These previous works now allow proceeding to the next step, that is, the inclusion of C/P functions aiming the development of double- and triple-ζ quality sets for general applications in relativistic electronic structure calculations of atomic and molecular systems.

In more details, each exponent for the Gaussian functions within the p-GCDF method, γ_*i*_^(*w*)^, for a given angular momentum symmetry, *w*, is generated through polynomial expansions truncated at third order,
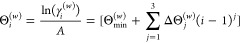
1and
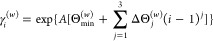
2being *A* = 6.0 and *i* = 1, 2,...,*N*, with *N* labeling the number of discretization points, while Θ_min_^(*w*)^ and ΔΘ_*q*_^(*w*)^ are, respectively, the starting point and the increment of order *q* needed to obtain the discretization points. In this context, the p-GCDF method stands out as a highly effective methodology for the generation of compact sets of Gaussian functions for relativistic calculations, which can be made prolapse-free by slight adjustments in the ΔΘ_1_^(*w*)^ parameter for the symmetries initially affected by this deficiency. In this approach, the polynomial parameters previously determined, employed for obtaining the primitive functions, can be reused for the production of supplementary functions, notably diffuse and polarization functions, which are considered in this study.

Hence, this work intends to provide Relativistic Prolapse-Free Gaussian basis sets of double- and triple-ζ quality (RPF-2Z and RPF-3Z, respectively) for all the atoms of the *s* and p blocks until Oganesson based on the SRPF and MRPF sets, respectively.^46−48^ A version of these basis sets augmented by extra diffuse functions for all symmetries (*s*, *p*, *d*, and *f*) is also provided (aug-RPF-2Z and aug-RPF-3Z). Some tests proved that these basis sets are comparable to other alternatives already developed of similar quality and may also result in some computational cost savings. However, although they are recommended for general calculations, these new prolapse-free basis sets must also stand out as a much better option for obtaining properties closely related to core electron distributions, for instance.

## 2 Methodology

The calculations discussed here were performed within the DIRAC 22 package.^49,50^ The Dirac-Coulomb (DC) Hamiltonian was chosen along with the Gaussian nuclear model and the standard light speed value of 137.0359992 atomic units (a.u.). An approximate treatment was employed to evaluate the interelectronic integrals among small functions (SS|SS),^51^ which are generated by kinetic balance conditions (upward and downward). Hence, the basis sets have always been used in their uncontracted versions.

The investigative calculations carried out to select correlation/polarization (C/P) functions are done by means of the multireferential configuration interaction method with single and double excitations (MR-CISD) of the valence space and were performed through the Relativistic Configuration Interaction Module within DIRAC 22. The Davidson’s correction was considered when available. In more details, the restricted active space (RAS) has been defined such that all excitations between valence electrons and valence spinors are allowed in RAS2, including as well up to double excitations to virtual spinors with energy up to 20.0 a.u. (RAS3). The RAS1 space was not considered in these calculations. The calculations for hydrogen and alkali metals were carried out for the ground state of MH molecules (M = hydrogen or an alkaline metal) in their equilibrium geometries from experiments,^52^ while a bond length value of 2.6 Å was assumed for FrH.

The basis set evaluation step took into account the investigation of the different properties. Thus, to this end, we considered DC calculations done at the Coupled Cluster level with single and double excitations, along with a perturbative treatment for triple substitutions (DC-CCSD-T). The equilibrium bond lengths (*r*_e_) and harmonic vibrational frequencies (ω_e_) were determined by fitting a fourth-order polynomial. The potential energy curve (PEC) was adjusted by considering the energy results from five points located around the minimum obtained by atomic displacements of 0.01 Å. Next, the electric dipole moments (μ) are calculated at the experimental equilibrium geometries. Namely, these quantities are determined by summing the analytical values of this property attained at the Dirac–Fock level with the electronic correlation contribution from DC-CCSD-T calculations, which are obtained by Response Theory with a proper perturbation and the finite difference technique with two points, that is
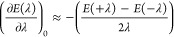
3Here, *E* represents the correlation energy and λ is the intensity of the field, defined as 1 × 10^–6^ a.u. The active space for calculations of molecular properties and electron affinities was defined in order to include all spinors with energies ranging from −5 to 20 a.u. Hence, the electron affinity was obtained by means of Fock Space calculations based on closed shell systems (anions for halogens and cations for group 13 elements) using the coupled pair theory with single and double substitutions (FS-CCSD).

## 3 Basis Set Augmentation with Correlation/Polarization Functions

The augmentation of basis sets with C/P functions requires the addition of carefully selected functions to deal properly with correlation/polarization effects in atomic and, mainly, molecular systems. Initially, these C/P functions must properly address the valence space of the elements considered. Here, we follow a choice procedure that resembles the one prescribed by Sadlej and co-workers,^53−55^ but using the functions already available with angular momentum *w –* 2 to achieve C/P functions of *w* angular momentum if this symmetry is still not present in the initial basis set. This approach will reduce the demands for computational resources because fewer small functions are generated by the kinetic balance conditions in such case. On the other hand, if functions of *w* symmetry are already included in the initial basis set, diffuse functions of that symmetry are evaluated for the C/P set.

In more details, the exponents of Gaussian functions in the *w* symmetry, γ_*i*_^(*w*)^, are obtained by means of the p-GCDF parameters of SRPFs and MRPFs corresponding to symmetry *w* – 2, which requires using the values of Θ_min_^(*w*–2)^ and ΔΘ_*q*_^(*w*–2)^ in accordance with eqs 1 and 2. Moreover, if diffuse functions are needed for the *w* symmetry, the parameters Θ_min_^(*w*)^ and ΔΘ_*q*_^(*w*)^ are employed in extrapolations done with eqs 1 and 2 (*i* = 0, −1, −2, and so on). Hence, the calculations for selecting the best *i* values were performed using the MR-CISD method, evaluating the effects of each function on the correlation energy.

Hence, RPF-2Z and aug-RPF-2Z basis sets are built based on primitive functions and p-GCDF parameters developed for the SRPF sets. In addition, RPF-3Z and aug-RPF-3Z basis sets are done starting from functions and parameters of the MRPF sets.^46−48^

### 3.1 Argon Example

Considering the RPF-2Z sets, based on SRPFs that already contain Gaussian functions of *s* and *p* symmetries for argon, the traditional double-ζ augmentation requires including one *d* function as the C/P set. As mentioned before, to reduce the number of small component functions, the exponents of *d* functions are generated from the parameters of the *s* symmetry, Θ_min_^(*s*)^ and ΔΘ_*q*_^(*s*)^. Hence, some *d* functions are individually considered, being labeled by their respective *i* values, which requires a comparison of their energetic effects from the MR-CISD calculations performed. According to Table 1, the *d* function associated with *i* = 2, *d*_2_, results in the lowest energy found. In other words, more diffuse or more compact *d* functions than *d*_2_ are less effective in describing the correlation of valence electrons within this atom. Thus, *d*_2_ is chosen to compose the C/P set of RPF-2Z for argon.

**Table 1:** Augmentation with C/P Functions for the RPF-2Z Set of Ar

basis^a^	exponent	*E* (Hartree)^b^	Δ*E* (mHartree)
SRPF		–528.733642	
+*d*_1_	0.2159	–528.762367	–28.725
+*d*_2_^c^	0.6095	–528.843004	–109.362
+*d*_3_	1.5803	–528.791780	–58.138
+*d*_4_	3.8511	–528.737409	–3.767
+*d*_5_	9.0273	–528.733701	–0.059

a The original SRPF is incremented by *d* functions with exponents (γ_*i*_^(*w*)^) given by the Θ_min_^(s)^ and ΔΘ_*q*_^(s)^ parameters (see eqs 1 and 2).

b Energies from MR-CISD calculations.

c Functions chosen to compose the final C/P set (the subscript represents the *i* value for the function).

C/P sets for triple-ζ basis sets of lighter p-block elements usually consist of two *d* functions and one *f* function. Thus, considering argon again as an example, Table 2 shows the augmentation process of MRPF in order to achieve the RPF-3Z set. First, the energetic effect of individual *d* functions with exponents provided by Θ_min_^(s)^ and ΔΘ_*q*_^(s)^ parameters is evaluated, showing that *d*_*2*_ and *d*_*3*_ produce the largest energy decrements. In a second moment, the combined effect of pairs of *d* functions is also analyzed, providing the decisive criterion for selection adopted here for the C/P sets. Again, the pair formed by functions *d*_2_ and *d*_3_ provides the best result. Subsequently, by starting from the MRPF set augmented by these two *d* functions, a study was carried out by individually adding *f* functions with exponents derived from Θ_min_^(p)^ and ΔΘ_*q*_^(p)^ parameters, which led to the choice of *f*_3_.

**Table 2:** Augmentation with C/P Functions for the RPF-3Z Set of Ar

basis^a^	exponent	*E* (Hartree)^b^	Δ*E* (mHartree)
MRPF		–528.736101	
+*d*_1_	0.1904	–528.757691	–21.590
+*d*_2_^c^	0.4955	–528.833468	–97.367
+*d*_3_^c^	1.1997	–528.823053	–86.952
+*d*_4_	2.7513	–528.749961	–13.860
+*d*_5_	6.0830	–528.736381	–0.280
+2*d*(*d*_1_, *d*_2_)		–528.837588	–101.487
+2*d*(*d*_1_, *d*_3_)		–528.854537	–118.436
+2*d*(*d*_1_, *d*_4_)		–528.777813	–41.712
+2*d*(*d*_2_, *d*_3_)		–528.872664	–136.562
+2*d*(*d*_2_, *d*_4_)		–528.858099	–121.998
+2*d*(*d*_3_, *d*_4_)		–528.825803	–89.702
MRPF + 2*d*		–528.872664	
+*f*_1_	0.1319	–528.874421	–1.757
+*f*_2_	0.3599	–528.890578	–17.915
+*f*_3_^c^	0.8899	–528.910799	–38.135
+*f*_4_	2.0643	–528.890458	–17.794
+*f*_5_	4.6487	–528.874270	–1.606

a The original MRPF is incremented by *d* functions with exponents (γ_*i*_^(*w*)^) given by the Θ_min_^(*s*)^ and ΔΘ_*q*_^(*s*)^ parameters and by *f* functions with exponents given by Θ_min_^(*p*)^ and ΔΘ_*q*_^(*p*)^ parameters (see eqs 1 and 2).

b Energies from MR-CISD calculations.

c Functions chosen to compose the final C/P set (the subscript represents the *i* value for the function).

Thus, the final RPF-2Z set of argon is composed by the original SRPF supplemented with the 1*d* function [γ^(*d*)^ = 0.6095], while the RPF-3Z set of this element is composed by the MRPF set augmented with 2*d* [γ^(*d*)^ = 0.4955 and 1.1997] and 1*f* [γ^(*f*)^ = 0.8899] functions. This C/P set for RPF-3Z of argon is correlation-consistent, with the effect of the second *d* function (−39.20 mHartree) being quite similar to the energetic effect of the first *f* function (−38.14 mHartree).

### 3.2 RPF-2Z and RPF-3Z Sets: *p*-Block Elements

First, the RPF-2Z sets derived from SRPFs^46−48^ are discussed in Tables 3, 4, and 5. As one can see, the *i* values for the additional *d* function of the C/P set tend to be maintained along each sub-block (2*p* and 3*p*), increasing in one unit only as going from boron to carbon, which is not surprising considering the atomic number increment along the period. This pattern illustrates an advantage of the polynomials employed in basis set development, which usually provide well-behaved trends of *i* values over the periods. As mentioned before, a diffuse *d* function is included as C/P set for 4*p*–7*p* sub-block elements (*i* = 0), which already contain occupied *d* spinors. Similarly to the RPF-4Z sets,^43^ a diffuse *s* function is also added for the 7*p* sub-block elements.

**Table 3:** Values of *i* That Define the C/P Functions for the RPF-2Z Set of 2*p* and 3*p* Elements According to Eqs 1 and 2 and to Their p-GCDF Parameters^46−48^

*w*	B	C	N	O	F	Ne	Al	Si	P	S	Cl	Ar
*d*^a^	2	3	3	3	3	3	2	2	2	2	2	2

a The Θ_min_^(*s*)^ and ΔΘ_*q*_^(*s*)^ parameters are used to obtain the *d* function exponents.

**Table 4:** Values of *i* That Define the C/P Functions for the RPF-2Z Set of 4*p* and 5*p* Elements According to Eqs 1 and 2 and to Their p-GCDF Parameters^46−48^

*w*	Ga	Ge	As	Se	Br	Kr	In	Sn	Sb	Te	I	Xe
*d*^a^	0	0	0	0	0	0	0	0	0	0	0	0

a The Θ_min_^(*d*)^ and ΔΘ_*q*_^(*d*)^ parameters are used to obtain the *d* function exponents.

**Table 5:** Values of *i* That Define the C/P Functions for the RPF-2Z Set of 6*p* and 7*p* Elements According to Eqs 1 and 2 and to Their p-GCDF Parameters^46−48^

*w*	Tl	Pb	Bi	Po	At	Rn	Nh	Fl	Mc	Lv	Ts	Og
*s*^a^							0	0	0	0	0	0
*d*^b^	0	0	0	0	0	0	0	0	0	0	0	0

a The Θ_min_^(*s*)^ and ΔΘ_*q*_^(*s*)^ parameters are used to obtain these function exponents.

b The Θ_min_^(*d*)^ and ΔΘ_*q*_^(*d*)^ parameters are used to obtain these function exponents.

Next, the RPF-3Z basis sets built based on MRPFs^46−48^ are discussed in Tables 6–8. As mentioned before, C/P sets for triple-ζ basis sets of lighter *p*-block elements are generally composed by two *d* and one *f* functions. Again, as evidenced in Table 6, the *i* values that provide the C/P functions are almost the same across each sub-block. The only exception occurs for Ne, which requires a larger *i* value for one *d* function due to the largest atomic number of this element along the 2*p* sub-block. In any case, the final C/P sets for 2*p* and 3*p* sub-blocks correspond to [2*d*,1*f*] functions. Thus, following to 4*p*–7*p* elements (see Tables 7 and 8), which already contain occupied *d* spinors, a single diffuse *d* function was added to their C/P sets. Of course, one *f* function was also included in these elements. Table 7 shows again the similar well-behaved pattern of *i* values observed before along sub-blocks, except for Xe, which requires an even more compact *f* function due to its larger atomic number compared to other 5*p* elements. Therefore, the final C/P set for 4*p*–6*p* elements is [1*d*,1*f*]. As done for the RPF-4Z sets,^43^ one diffuse *s* function was also required for 7*p* elements, [1*s*,1*d*,1*f*].

**Table 6:** Values of *i* That Define the C/P Functions for the RPF-3Z Set of 2*p* and 3*p* Elements According to Eqs 1 and 2 and to Their p-GCDF Parameters^46−48^

*w*	B	C	N	O	F	Ne	Al	Si	P	S	Cl	Ar
*d*^a^	2,3	2,3	2,3	2,3	2,3	2,4	2,3	2,3	2,3	2,3	2,3	2,3
*f*^b^	4	4	4	4	4	4	3	3	3	3	3	3

a The Θ_min_^(*s*)^ and ΔΘ_*q*_^(*s*)^ parameters are used to obtain these function exponents.

b The Θ_min_^(*p*)^ and ΔΘ_*q*_^(*p*)^ parameters are used to obtain these function exponents.

**Table 7:** Values of *i* That Define the C/P Functions for the RPF-3Z Set of 4*p* and 5*p* Elements According to Eqs 1 and 2 and to Their p-GCDF Parameters^46−48^

*w*	Ga	Ge	As	Se	Br	Kr	In	Sn	Sb	Te	I	Xe
*d*^a^	0	0	0	0	0	0	0	0	0	0	0	0
*f*^b^	3	3	3	3	3	3	2	2	2	2	2	3

a The Θ_min_^(*d*)^ and ΔΘ_*q*_^(*d*)^ parameters are used to obtain these function exponents.

b The Θ_min_^(*p*)^ and ΔΘ_*q*_^(*p*)^ parameters are used to obtain these function exponents.

**Table 8:** Values of *i* That Define the C/P Functions for the RPF-3Z Set of 6*p* and 7*p* Elements According to Eqs 1 and 2 and to Their p-GCDF Parameters^46−48^

*w*	Tl	Pb	Bi	Po	At	Rn	Nh	Fl	Mc	Lv	Ts	Og
*s*^a^							0	0	0	0	0	0
*d*^b^	0	0	0	0	0	0	0	0	0	0	0	0
*f*^c^	0	0	0	0	0	0	0	0	0	0	0	0

a The Θ_min_^(*s*)^ and ΔΘ_*q*_^(*s*)^ parameters are used to obtain these function exponents.

b The Θ_min_^(*d*)^ and ΔΘ_*q*_^(*d*)^ parameters are used to obtain these function exponents.

c The Θ_min_^(*f*)^ and ΔΘ_*q*_^(*f*)^ parameters are used to obtain these function exponents.

### 3.3 RPF-2Z and RPF-3Z Sets: *s*-Block Elements

Some of the *s*-block elements present a particularity once they are in the imminence for the initial filling of spinors of larger angular momentum than the ones already occupied before. For instance, lithium and beryllium precede the 2*p* sub-block. Hence, these elements must include a reasonable number of *p* functions to give a proper description of their valence properties. The same occurs for potassium and calcium, which will require some *d* functions, and for cesium and barium, in the case of *f* functions. Based on the RPF-4Z sets already developed as an upper limit for this augmentation process,^43^ 6*p* and 7*p* functions are selected, respectively, for RPF-2Z and RPF-3Z sets of Li and Be. Moreover, by similar arguments, 5*d* and 6*d* functions are added, respectively, to RPF-2Z and RPF-3Z sets of K and Ca, while 2*f* and 3*f* functions are inserted, respectively, into RPF-2Z and RPF-3Z sets of Cs and Ba. The functions chosen for this end are added sequentially (preserving the previous functions in that symmetry, as new ones are included), starting from an energetically significant function in the diffuse region (*i* = 0 or 1). As also noticed before,^43^ some of these atoms also need two diffuse *p* functions (from Na to Ra) and one or two diffuse *d* functions (from Rb to Ra) for better valence space descriptions. This occurs because previously occupied spinors of such symmetries now lie in the core region of these elements, and more diffuse *p* and *d* functions are now energetically justified.

In this paragraph, the RPF-2Z sets derived from SRPFs^46−48^ are discussed. As expected, the C/P set for H and He will be constituted by one *p* function (*p*_3_), as indicated in Table 9. Regarding the 2*s* elements, Li and Be, along with the 6*p* functions mentioned before (*p*_1_ - *p*_6_), one *d* function (*d*_3_) is added for polarization reasons, leading to a [6*p*,1*d*] C/P set. One *d* function was also selected for polarization in Na and Mg (*d*_2_), providing a [2*p*,1*d*] C/P set as one considers the diffuse *p* functions previously included. For K and Ca, as discussed before, the C–P set encompasses [2*p*,5*d*] functions. Additionally, for Rb and Sr, the C/P set is provided by the diffuse functions cited before, [2*p*,2*d*]. Finally, according to the previous comments, Cs and Ba will require a [2*p*,2*d*,2*f*] C/P set, while Fr and Ra are augmented by [2*p*,1*d*] diffuse functions.

**Table 9:** Values of *i* That Define the C/P Functions for the RPF-2Z Set of *s*-Block Elements According to Eqs 1 and 2 and to Their p-GCDF Parameters^46−48^

*w*	H	He	Li	Be	Na	Mg	K	Ca	Rb	Sr	Cs	Ba	Fr	Ra
*p*	3^a^	3^a^	1–6^a^	1–6^a^	–1,0^b^	–1,0^b^	–1,0^b^	–1,0^b^	–1,0^b^	–1,0^b^	–1,0^b^	–1,0^b^	–1,0^b^	–1,0^b^
*d*			3^a^	3^a^	2^a^	2^a^	1–5^a^	1–5^a^	–1,0^c^	–1,0^c^	–1,0^c^	–1,0^c^	0^c^	0^c^
*f*											1,2^b^	0,1^b^		

a The Θ_min_^(*s*)^ and ΔΘ_*q*_^(*s*)^ parameters are used to obtain these function exponents.

b The Θ_min_^(*p*)^ and ΔΘ_*q*_^(*p*)^ parameters are used to obtain these function exponents.

c The Θ_min_^(*d*)^ and ΔΘ_*q*_^(*d*)^ parameters are used to obtain these function exponents.

Next, the RPF-3Z sets provided by the augmentation of MRPFs^46−48^ are considered in Table 10. As is customary during a triple-ζ polarization for H and He, the C/P set of these elements is constituted by [2*p*,1*d*] functions. As mentioned before, the C/P sets for Li and Be require 7*p* as well as 2*d* and 1*f* functions, [7*p*,2*d*,1*f*]. Next, Na and Mg initially require two diffuse *p* functions, augmented by 2*d* and 1*f* functions for polarization, resulting in a [2*p*,2*d*,1*f*] C/P set. The C/P set for K and Ca comprises two diffuse *p* and 6*d* functions, as commented on earlier, as well as 1*f* function, [2*p*,6*d*,1*f*]. In the sequence, Rb and Sr possess a C/P set including two diffuse *p* and *d* functions, supplemented by a 1*f* Gaussian, [2*p*,2*d*,1*f*]. Finally, the C/P set of Cs and Ba also considers two diffuse *p* and *d* functions, with the subsequent addition of 3*f* functions, [2*p*,2*d*,3*f*], while a C/P set of [2*p*,2*d*,2*f*] diffuse functions is selected for Fr and Ra.

**Table 10:** Values of *i* That Define the C/P Functions for the RPF-3Z Set of *s*-Block Elements According to Eqs 1 and 2 and to Their p-GCDF Parameters^46−48^

*w*	H	He	Li	Be	Na	Mg	K	Ca	Rb	Sr	Cs	Ba	Fr	Ra
*p*	3,4^a^	2,3^a^	1–7^a^	1–7^a^	–1,0^b^	–1,0^b^	–1,0^b^	–1,0^b^	–1,0^b^	–1,0^b^	–1,0^b^	–1,0^b^	–1,0^b^	–1,0^b^
*d*	3^a^	3^a^	2,3^a^	2,3^a^	2,4^a^	2,3^a^	1–6^a^	1–6^a^	–1,0^c^	–1,0^c^	–1,0^c^	–1,0^c^	–1,0^c^	–1,0^c^
*f*			3^a^	3^a^	0^b^	0^b^	0^b^	1^b^	1^b^	1^b^	0–2^b^	0–2^b^	–1,0^d^	–1,0^d^

a The Θ_min_^(*s*)^ and ΔΘ_*q*_^(*s*)^ parameters are used to obtain these function exponents.

b The Θ_min_^(*p*)^ and ΔΘ_*q*_^(*p*)^ parameters are used to obtain these function exponents.

c The Θ_min_^(*d*)^ and ΔΘ_*q*_^(*d*)^ parameters are used to obtain these function exponents.

d The Θ_min_^(*f*)^ and ΔΘ_*q*_^(*f*)^ parameters are used to obtain these function exponents.

### 3.4 aug-RPF-2Z and aug-RPF-3Z Sets

The previous basis sets are also augmented by adding one more diffuse function to every orbital symmetry already present (*s*, *p*, *d*, and *f*) in order to achieve aug-RPF-2Z and aug-RPF-3Z sets. The exponents of these diffuse functions are obtained by using *i* values one unit smaller than the smallest ones already considered in each symmetry for the RPF-2Z and RPF-3Z sets.

## 4 Molecular and Atomic Tests

In this section, we first address the results obtained from DC-CCSD-T molecular calculations using the RPF-2Z and RPF-3Z basis sets for equilibrium distances (*r*_e_), harmonic vibrational frequencies (ω_e_), and dipole moments (μ). These data are also compared with those obtained from dyall.v2z and dyall.v3z sets.^31−35^ The analysis covers simple diatomic molecules, such as MH (M = Li, Na, K, Rb, and Cs), HX (X = F, Cl, Br, and I), and YF (Y = B, Al, Ga, In, and Tl). Additionally, electron affinities (EAs) of halogen and group 13 atoms were determined using the augmented versions of our basis sets that include additional diffuse functions (aug-RPF-2Z and aug-RPF-3Z) through FS-CCSD calculations. The EAs obtained are compared with those from dyall.av2z and dyall.av3z sets.^31−34^ The mean absolute deviations (MADs) and maximum absolute errors (MAEs) achieved with respect to experimental data^52,56^ are presented in Table 11. As expected, there is a clear pattern of improvement in the overall results as the quality of the basis set increases. Furthermore, the results from the (aug-)RPF-2Z and (aug-)RPF-3Z sets are comparable with those from dyall.(a)v2z and dyall.(a)v3z sets, respectively. However, our basis sets are now free of variational prolapse.

**Table 11:** Mean Absolute Deviations (MADs) and Maximum Absolute Errors (MAEs) Determined in DC-CCSD-T (for *r*_e_, ω_e_, and μ)^a^ and FS-CCSD Calculations (for EAs)^a^ with Respect to Experimental Data^52,56^

basis sets	*r*_e_ (Å)		ω_e_ (cm^–1^)		μ (D)^b^		EA (eV)^c^	
	MAD	MAE	MAD	MAE	MAD	MAE	MAD	MAE
RPF-2Z	0.0204	0.0541	32	97	0.10	0.21	0.15	0.25
RPF-3Z	0.0073	0.0185	13	42	0.08	0.22	0.10	0.15
dyall.v2z	0.0216	0.0600	26	82	0.12	0.35	0.15	0.22
dyall.v3z	0.0078	0.0217	10	25	0.07	0.20	0.10	0.16

a The following molecules are considered for *r*_e_, ω_e_, and μ calculations: MH (M = Li, Na, K, Rb, and Cs), HX (X = F, Cl, Br, and I), and YF (Y = B, Al, Ga, In, and Tl). Halogens and group 13 atoms are considered for EA determinations.

b NaH, KH, RbH, CsH, and BF are not included in the error determinations due to the lack of accurate experimental data.

c This property was determined by using the respective basis sets in their augmented versions with diffuse functions (aug-RPF-XZ and dyall.avxz).

Table 12 shows the CPU times for CCSD-T calculations done at fixed geometries of the molecular systems investigated here. As one can see, RPF-2Z sets usually require a little longer CPU times than dyall.v2z. However, the RPF-3Z sets are almost always less demanding than dyall.v3z. Hence, the elimination of variational prolapse can also provide efficient basis sets in terms of computational resources.

**Table 12:** Total CPU Time in DC–CCSD-T Calculations with Different Basis Sets Performed in an Intel(R) Xeon(R) Gold 5318Y CPU @ 2.10 GHz Processor

time (s)	RPF-2Z	RPF-3Z	dyall.v2z	dyall.v3z
LiH	6	23	4	19
NaH	6	27	8	129
KH	25	101	11	188
RbH	66	231	56	807
CsH	241	641	101	1101
HF	5	19	4	22
HCl	5	24	5	28
HBr	38	120	16	149
HI	64	176	40	296
BF	15	101	10	119
AlF	22	150	12	167
GaF	117	486	57	597
InF	176	790	124	1094
TlF	1115	3362	680	3910

## 5 Conclusions

In this study, Correlation/Polarization (C/P) sets were incorporated into SRPFs and MRPFs by means of a choice process guided by MR-CISD calculations of valence electrons in order to provide double- and triple-ζ quality basis sets without variational prolapse for all elements of s and p blocks (RPF-2Z and RPF-3Z). Additionally, versions of these sets with extra diffuse functions for each available symmetry are also generated (aug-RPF-2Z and aug-RPF-3Z). Hence, the augmentation procedure with more diffuse functions or with functions from higher angular momentum symmetries is made simpler by the characteristics of the p-GCDF method. Results from molecular and atomic calculations of fundamental properties confirm the quality expected from (aug-)RPF-2Z and (aug-)RPF-3Z sets, which may also exhibit computational efficiency advantages in certain cases. Finally, the prolapse-free design of these new basis sets recommends their usage in calculations of properties more dependent on inner core electron distributions. The basis sets developed here are available as separated text files in the Supporting Information Material.

## Supporting Information Available

Equilibrium bond lengths (Å) obtained at the DC-CCSD-T level; harmonic vibrational frequencies (cm^−1^) obtained at the DC-CCSD-T level; molecular dipole moments (*D*) obtained at the DC-CCSD-T level; and electron affinities (eV) obtained at the FS-CCSD level (PDF)
Relativistic prolapse-free Gaussian basis sets of double-zeta quality: RPF-2Z (TXT)
Relativistic prolapse-free Gaussian basis sets of triple-zeta quality: RPF-3Z (TXT)
Augmented relativistic prolapse-free Gaussian basis sets of double-zeta quality: aug-RPF-2Z (TXT)
Augmented relativistic prolapse-free Gaussian basis sets of triple-zeta quality: aug-RPF-3Z (TXT)
