# Supplementary material for: Relativistic Prolapse-Free Gaussian Basis Sets of Double- and Triple-ζ Quality for s- and p-Block Elements: (aug-)RPF-2Z and (aug-)RPF-3Z
Source: J Chem Theory Comput. 2024 Nov 14;20(22):9991–8. doi: 10.1021/acs.jctc.4c01211 (PMC11603607; doi:10.1021/acs.jctc.4c01211)
Supplement: Supplementary file 1 — ct4c01211_si_001.pdf [file ct4c01211_si_001.pdf]

Relativistic Prolapse-Free Gaussian Basis Sets of Double- and Triple-ζ Quality for s- and p-Block Elements: (aug-)RPF-2Z and (aug-)RPF-3Z

Julielson dos Santos Sousa,* Eriosvaldo Florentino Gusmão,* Anne Kéllen de Nazaré dos Reis Dias,* and Roberto Luiz Andrade Haiduke*

Department of Chemistry and Molecular Physics, São Carlos Institute of Chemistry, University of São Paulo, C.P. 780, 13560-970, São Carlos, SP,Brazil.

E-mail: julielson@usp.br; eriosvaldo.gusmao@alumni.usp.br; annekellenreis@usp.br; haiduke@iqsc.usp.br

Supporting Information

Table S1: Equilibrium bond lenghts (Å) obtained at the DC-CCSD-T level.[a]

Molecule	RPF-2Z	RPF-3Z	dyall.v2z	dyall.v3z	Exp.[b]
^{1}LiH	1.5872	1.5932	1.5925	1.5964	1.5957
^{23}NaH	1.9068	1.8841	1.8826	1.8860	1.8874
^{39}KH	2.2705	2.2568	2.2661	2.2523	2.2425
^{85}RbH	2.3942	2.3716	2.3891	2.3702	2.367
^{133}CsH	2.5013	2.4909	2.5455	2.5082	2.4938
H^{19}F	0.9151	0.9166	0.9209	0.9187	0.916808
H^{35}Cl	1.2846	1.2776	1.2885	1.2783	1.274552
H^{81}Br	1.4106	1.4127	1.4197	1.4141	1.414435
H^{127}I	1.6088	1.6051	1.6150	1.6121	1.60916
^{11}B^{19}F	1.2834	1.2745	1.2797	1.2710	1.26259
^{27}Al^{19}F	1.7085	1.6702	1.7143	1.6761	1.654369
^{69}Ga^{19}F	1.7995	1.7816	1.7990	1.7848	1.774369
^{115}In^{19}F	2.0284	2.0039	2.0225	2.0046	1.985396
^{205}Tl^{19}F	2.1205	2.0968	2.1128	2.0948	2.084438
**MAD**[c]	**0.0204**	**0.0073**	**0.0216**	**0.0078**	
**MAE**[d]	**0.0541**	**0.0185**	**0.0600**	**0.0217**	

[a] Bond length results are determined through a fourth order polynomial adjustment given by five energy values around the minimum in the potential energy curve (see the main text);.
[b] These values are taken from the NIST Standard Reference Data [Klaus P. Huber and Gerhard H. Herzberg (data prepared by Jean W. Gallagher and Russell D. Johnson, III) "Constants of Diatomic Molecules" in NIST Chemistry WebBook, NIST Standard Reference Database Number 69, Eds. P.J. Linstrom and W.G. Mallard, National Institute of Standards and Technology, Gaithersburg MD, 20899, https://doi.org/10.18434/T4D303 (accessed Jul 17, 2024)];
[c] Mean absolute deviation with respect to the experimental values;
[d] Maximum absolute error with respect to the experimental values.

Table S2: Harmonic vibrational frequencies (cm^{-1}) obtained at the DC-CCSD-T level.[a]

Molecule	RPF-2Z	RPF-3Z	dyall.v2z	dyall.v3z	Exp.[b]
^{1}LiH	1431	1421	1419	1409	1405.65
^{23}NaH	1149	1183	1185	1183	1172.2
^{39}KH	974	974	986	991	983.6
^{85}RbH	915	949	928	944	936.94
^{133}CsH	900	896	861	893	891.0
$H^{19}F$	4190	4180	4095	4163	4138.32
$H^{35}Cl$	3009	3002	3001	3010	2990.9463
$H^{81}Br$	2680	2685	2680	2674	2648.975
$H^{127}I$	2298	2316	2330	2321	2309.014
$^{11}B^{19}F$	1306	1385	1336	1393	1402.13
$^{27}Al^{19}F$	737	803	720	793	802.26
$^{69}Ga^{19}F$	595	627	605	620	622.2
$^{115}In^{19}F$	505	523	521	533	533.35
$^{205}Tl^{19}F$	450	470	468	469	477.3
**MAD**[c]	**32**	**13**	**26**	**10**	
**MAE**[d]	**97**	**42**	**82**	**25**	

[a] Harmonic vibrational frequency results are determined through a fourth order polynomial adjustment given by five energy values around the minimum in the potential energy curve (see the main text);.

[b] These values are taken from the NIST Standard Reference Data [Klaus P. Huber and Gerhard H. Herzberg (data prepared by Jean W. Gallagher and Russell D. Johnson, III) "Constants of Diatomic Molecules" in NIST Chemistry WebBook, NIST Standard Reference Database Number 69, Eds. P.J. Linstrom and W.G. Mallard, National Institute of Standards and Technology, Gaithersburg MD, 20899, https://doi.org/10.18434/T4D303 (accessed Jul 17, 2024)];

[c] Mean absolute deviation with respect to the experimental values;

[d] Maximum absolute error with respect to the experimental values.

Table S3: Molecular dipole moments (D) obtained at the DC-CCSD-T level.[a]

Molecule	RPF-2Z	RPF-3Z	dyall.v2z	dyall.v3z	Exp.[b]
^{1}LiH	5.72	5.79	5.78	5.83	5.884
^{23}NaH	6.38	6.35	6.23	6.36	
^{39}KH	7.85	8.00	7.92	8.01	
^{85}RbH	7.93	8.04	8.11	8.19	
^{133}CsH	8.03	8.17	8.28	8.33	
H^{19}F	1.95	1.88	1.85	1.80	1.826178
H^{35}Cl	1.32	1.23	1.19	1.12	1.1086
H^{81}Br	0.91	0.86	0.88	0.84	0.8272
H^{127}I	0.50	0.47	0.51	0.46	0.448
^{11}B^{19}F	0.79	0.86	0.85	0.87	0.5
^{27}Al^{19}F	1.59	1.43	1.53	1.40	1.53
^{69}Ga^{19}F	2.41	2.35	2.32	2.31	2.45
^{115}In^{19}F	3.30	3.18	3.19	3.20	3.40
^{205}Tl^{19}F	4.29	4.22	4.15	4.19	4.2282
**MAD**[c]	**0.10**	**0.08**	**0.12**	**0.07**	
**MAE**[d]	**0.21**	**0.22**	**0.35**	**0.20**	

[a] These results were obtained at the experimental equilibrium ground state geometry [Klaus P. Huber and Gerhard H. Herzberg (data prepared by Jean W. Gallagher and Russell D. Johnson, III) "Constants of Diatomic Molecules" in NIST Chemistry WebBook, NIST Standard Reference Database Number 69, Eds. P.J. Linstrom and W.G. Mallard, National Institute of Standards and Technology: Gaithersburg MD, 20899, https://doi.org/10.18434/T4D303 (accessed Jul 17, 2024)] by the sum of the analytic DFC value with DC-CCSD-T electron correlation contributions given by the finite-difference technique in a two point approach (see the main text);
[b] These values are taken from the CRC Handbook of Chemistry and Physics [CRC Handbook of Chemistry and Physics, 94th ed.; Internet Version 2014; Ed. W. M. Haynes, CRC Press/Taylor and Francis: Boca Raton, FL, 2014; Available online: http://www.hbcpnetbase.com/ (accessed May 15, 2014).];
[c] Mean absolute deviation with respect to the experimental values;
[d] Maximum absolute error with respect to the experimental values.

Table S4: Electron affinities (eV) obtained at the FS-CCSD level.

Atom	aug-RPF-2Z	aug-RPF-3Z	dyall.av2z	dyall.av3z	Exp.[a]
F	3.1528	3.2851	3.1857	3.3040	3.4011897
Cl	3.4435	3.5549	3.4664	3.5524	3.612725
Br	3.2095	3.2890	3.2159	3.2907	3.3635882
I	2.8817	2.9359	2.8809	2.9615	3.0590368
B	0.0880	0.1336	0.1074	0.1338	0.279723
Al	0.3443	0.3782	0.3397	0.3753	0.43283
Ga	0.2387	0.2796	0.2328	0.2718	0.43
In	0.3238	0.3648	0.3204	0.3685	0.3
Tl	0.2427	0.2657	0.2005	0.2627	0.377
**MAD**[b]	**0.15**	**0.10**	**0.15**	**0.10**	
**MAE**[c]	**0.25**	**0.15**	**0.22**	**0.16**	

[a] These values are taken from the CRC Handbook of Chemistry and Physics [CRC Handbook of Chemistry and Physics, 94th ed.; Internet Version 2014; Ed. W. M. Haynes, CRC Press/Taylor and Francis: Boca Raton, FL, 2014; Available online: http://www.hbcpnetbase.com/ (accessed May 15, 2014).];
[b] Mean absolute deviation with respect to the experimental values;
[c] Maximum absolute error with respect to the experimental values.
